# *Trypanosoma cruzi* in Nonischemic Cardiomyopathy Patients, Houston, Texas, USA

**DOI:** 10.3201/eid2707.203244

**Published:** 2021-07

**Authors:** Melissa S. Nolan, David Aguilar, Arunima Misra, Sarah M. Gunter, Tim Erickson, Rodion Gorchakov, Hilda Rivera, Susan P. Montgomery, Kristy O. Murray

**Affiliations:** University of South Carolina, Columbia, South Carolina, USA (M.S. Nolan);; Baylor College of Medicine, Houston, Texas, USA (M.S. Nolan, D. Aguilar, A. Misra, S.M. Gunter, T. Erickson, R. Gorchakov, K.O. Murray);; Harris Health System–Ben Taub Hospital, Houston (A. Misra);; Centers for Disease Control and Prevention, Atlanta, Georgia, USA (H. Rivera, S.P. Montgomery)

**Keywords:** *Trypanosoma cruzi*, Chagas disease, Houston, Texas, USA, cardiomyopathy, surveillance, parasites

## Abstract

To investigate possible cardiac manifestations of Chagas disease, we tested 97 Latinx patients with nonischemic cardiomyopathy in Houston, Texas, USA, for *Trypanosoma cruzi* infection. We noted a high prevalence of underdiagnosed infection and discrepant results in clinical diagnostic assays. Latinx cardiac patients in the United States would benefit from laboratory screening for *T. cruzi* infection.

The clinical manifestations of Chagas disease, caused by infection with the *Trypanosoma cruzi* parasite, are cardiac in approximately one third of patients. Without treatment, the parasite alternates between the trypomastigote and amastigote forms and causes direct smooth muscle tissue damage, myocardial fibrosis, chronic activation of inflammatory pathways, and autonomic dysfunction (*1*). This process can lead to progressive heart failure years later for some patients. Chagas cardiomyopathy patients can seek treatment for malignant ventricular arrhythmias, aneurysms, thromboembolism, or sudden cardiac death (*2*). Despite advances in our understanding of the pathogenic pathways, why some patients have onset of progressive cardiac disease whereas others remain in a persistent subclinical indeterminate disease remain unknown. Identifying infection status early, before the onset of heart failure, is critical because chemotherapeutics are most efficacious in the acute and early stages of infection.

In the United States, ≈300,000 persons are infected with *T. cruzi* parasites (*3*), and <1% have received treatment (*4*). Because of low physician awareness (*5*), Chagas disease often is underdiagnosed or misdiagnosed. Previous cardiac patient seroprevalence studies in New York, NY, and Los Angeles, CA, suggest that the rate of undiagnosed *T. cruzi* infection is particularly high (13%–19%) among Latin American immigrants with dilated cardiomyopathy (*6*,*7*). However, the extent of *T. cruzi* infection in the United States beyond these 2 metropolitan areas is largely unknown. We assessed the utility of *T. cruzi* diagnostic surveillance for Latinx patients with nonischemic cardiomyopathy who sought clinical care in a large tertiary care facility in Houston, Texas, USA.

## The Study

During August 2015–July 2017, we recruited cardiac patients for Chagas disease surveillance from Harris Health System–Ben Taub Hospital, a large county-funded tertiary care facility in Houston. Patients with known nonischemic cardiomyopathy who sought treatment at the outpatient cardiac clinic or who were admitted to a cardiac inpatient unit were invited to participate in our study. Inclusion criteria required a recorded ejection fraction <50% within the past year and a recent negative ischemic work-up based on stress echocardiography or invasive coronary angiography. We excluded patients of non-Latinx ethnicity and those who were currently incarcerated, had prior *T. cruzi* serologic testing, had evidence of acute coronary syndrome suspected to be of Takotsubo origin, or had documentation of an alternative etiology for their nonischemic cardiomyopathy (e.g., peripartum, genetic, or alcoholic cardiomyopathy). Consent forms were available in English and Spanish, and licensed translators ensured that all potentially eligible participants were invited to participate. This protocol was reviewed and approved by the Baylor College of Medicine Institutional Review Board (protocol no. H-36761).

After consent, participating patients provided a blood sample for *T. cruzi* diagnostic testing and completed a risk factor questionnaire. The 5-page questionnaire was administered by a study team member and included sections on residential and travel histories, potential triatomine exposures and sources, current health symptoms and health behaviors, clinical family history, and knowledge, attitudes, and practices regarding Chagas disease. Initial *T. cruzi* diagnostic testing included *T. cruzi*–specific antibody testing using Chagas STAT-PAK Assay (Chembio Diagnostic Systems, Inc., https://chembio.com) and Hemagen Chagas Kit (Hemagen Diagnostics, Inc., https://www.hemagen.com). Confirmation of positive and discordant results were then performed by using Chagatest ELISA Recombinante 3.0 (Wiener Laboratorios S.A.I.C., https://www.wiener-lab.com) and TESA blot by the Centers for Disease Control and Prevention.

During the 2-year study period, 97 patients with nonischemic cardiomyopathy were enrolled out of 132 eligible patients; 35 refused to participate because of lack of interest. The average age of participants was 52 years (range 28–91 years); 38% of participants were female and 62% male. Birth countries for the cohort were Mexico (53%), United States (14%), El Salvador (12%), Honduras (9%), Guatemala (4%), and other Latin America Spanish-speaking countries (8%). Patients born in the United States originated from Texas (n = 9), New York (n = 2), Indiana (n = 1), and Oregon (n = 1). Of the cohort, 43% reported having previously seen the triatomine vector; 20/42 (48%) reported sightings in Texas, compared with 31/42 (74%) in a Chagas-endemic Latin American country. Furthermore, 12% of the cohort reported a history of triatomine bites. Despite high triatomine recognition, only 8% of the patient cohort had ever heard of Chagas disease, and only half of these patients could correctly state how Chagas disease is acquired.

Overall, 7% of Latinx nonischemic cardiomyopathy patients seeking treatment for heart failure management were confirmed positive for *T. cruzi* infection by Centers for Disease Control and Prevention Wiener EIA and TESA blot confirmation testing. Discordant test results were common (Table), complicating the clinical decision-making process. All 7 patients who had laboratory-confirmed Chagas cardiomyopathy were born in a Latin America country: El Salvador (n = 4), Honduras (n = 1), Mexico (n = 1), and Venezuela (n = 1). All 7 confirmed positive patients had mothers who were born in or had lived in a Latin America country. Three had lived in a house with a dirt floor and 2 with a palm leaf thatched roof, which are both known risks for triatomine infestations (*8*,*9*). One participant had received a blood transfusion in their home country. Two were polyparous mothers, and none of their children had been tested for Chagas disease. Only 2 of the 7 patients with Chagas cardiomyopathy had ever heard of Chagas disease, and only 1 of these patients knew how Chagas disease was acquired.

**Table:** Characteristics of patients enrolled in a cross-sectional study of *Trypanosoma cruzi* infections in Latinx cardiomyopathy patients at a tertiary care facility† and results of 4 diagnostic assays, Houston, Texas, USA, 2015–2017*

ID	Age, y/sex	State, country of birth	True positive†§	BCM testing			CDC testing	
				Stat-Pak	Hemagen		Weiner EIA	TESA blot
CM-013	79/F	Guerrero, Mexico	No	Faint positive	–		–	NP
CM-014	66/M	La Union, El Salvador	Yes	+	+		+	+
CM-017	62/M	San Salvador, El Salvador	Yes	+	+		+	+
CM-037	73/F	El Salvador‡	Yes	Faint positive	–		+	+
CM-048	54/M	Texas, USA	No	Faint Positive	–		–	NP
CM-058	68/F	Michoacan, Mexico	No	-	+		–	NP
CM-082	70/F	Tegucigalpa, Honduras	Yes	+	+		+	+
CM-116	34/M	Acapulco, Mexico	No	Faint positive	–		–	NP
CM-121	77/M	Maracay, Venezuela	Yes	+	+		+	+
CM-143	42/M	San Miguel, El Salvador	Yes	+	+		+	+
CM-155	73/M	Unreported‡	No	Faint positive	–		–	NP
CM-174	78/M	Guerrero, Mexico	Yes	+	+		+	+
CM-197	62/M	Tamaulipas, Mexico	No	+	–		–	NP
CM-243	54/M	Durango, Mexico	No	Faint positive	–		–	NP

*All patients were of White race and Latinx ethnicity. A total of 83 patients tested negative by STAT-PAK (Chembio Diagnostic Systems, Inc., https://chembio.com) and Hemagen (Hemagen Diagnostics, Inc., https://www.hemagen.com). This table displays the 14 patients who tested positive on >1 of the screener assays, whose samples where then sent to CDC for testing. None of the 83 patients who tested negative by the 2 screening assays had samples sent to CDC for confirmation testing. BCM, Baylor College of Medicine; CDC, Centers for Disease Control and Prevention; EIA, enzyme immunoassay; ID, identification; NP, not performed; –, negative; +, positive. †True positive refers to the CDC guidelines recommending a minimum of >2 positive test results using >2 different diagnostic assay techniques (https://www.cdc.gov/parasites/chagas/healthprofessionals/dx.html). ‡Participants choose not to answer state, country of birth, or both because of personal concerns.

## Conclusions

Our study adds to the growing body of evidence supporting *T. cruzi* surveillance of Latinx patients with nonischemic cardiomyopathy or other risk factors for *T. cruzi* infection in the United States. *T. cruzi* infection accounts for a considerable proportion of nonischemic cardiomyopathy in foreign-born Latinx patients (7%–19%) (*4*,*5*), and the timely diagnosis of their infection is imperative. 

Our investigation has a few limitations, including the inability to perform additional cardiac imaging and diagnostic studies or follow patients long-term to evaluate prospective identification of underlying etiology. As highlighted by our discordant results, further work is needed to develop a highly specific diagnostic test to prevent clinical confusion regarding accurate disease status. Determining the underlying etiology has a benefit for Chagas cardiomyopathy patients despite the limited efficacy of treatment with antiparasitics (benznidazole and nifurtimox). Patients with Chagas cardiomyopathy might be recommended for heart transplant (*10*) and can positively respond to implantable cardioverter-defibrillator placement (*11*) and amiodarone (*12*). Awareness of infection could lead to testing of at-risk family members who might respond favorably to early treatment.

## References

[1] Bern C, Messenger LA, Whitman JD, Maguire JH. Chagas disease in the United States: a public health approach. Clin Microbiol Rev. 2019;33:e00023-19. 10.1128/CMR.00023-19 31776135 PMC6927308

[2] Pino-Marín A, Medina-Rincón GJ, Gallo-Bernal S, Duran-Crane A, Arango Duque ÁI, Rodríguez MJ, Medina-Mur R, Manrique FT, Forero JF, Medina HM. Chagas cardiomyopathy: from Romaña sign to heart failure and sudden cardiac death. Pathogens. 2021 Apr 22;10(5):505 33922366 10.3390/pathogens10050505 PMC8145478

[3] Manne-Goehler J, Umeh CA, Montgomery SP, Wirtz VJ. Estimating the burden of Chagas disease in the United States. PLoS Negl Trop Dis. 2016;10:e0005033. 10.1371/journal.pntd.0005033 27820837 PMC5098725

[4] Manne-Goehler J, Reich MR, Wirtz VJ. Access to care for Chagas disease in the United States: a health systems analysis. Am J Trop Med Hyg. 2015;93:108–13. 10.4269/ajtmh.14-0826 25986581 PMC4497880

[5] Stimpert KK, Montgomery SP. Physician awareness of Chagas disease, USA. Emerg Infect Dis. 2010;16:871–2. 10.3201/eid1605.091440 20409389 PMC2953998

[6] Traina MI, Sanchez DR, Hernandez S, Bradfield JS, Labedi MR, Ngab TA, et al. Prevalence and impact of Chagas disease among Latin American immigrants with nonischemic cardiomyopathy in Los Angeles, California. Circ Heart Fail. 2015;8:938–43. 10.1161/CIRCHEARTFAILURE.115.002229 26206855 PMC10108371

[7] Kapelusznik L, Varela D, Montgomery SP, Shah AN, Steurer FJ, Rubinstein D, et al. Chagas disease in Latin American immigrants with dilated cardiomyopathy in New York City. Clin Infect Dis. 2013;57:e7. 10.1093/cid/cit199 23537911 PMC10123391

[8] Bustamante DM, De Urioste-Stone SM, Juárez JG, Pennington PM. Ecological, social and biological risk factors for continued *Trypanosoma cruzi* transmission by *Triatoma dimidiata* in Guatemala. PLoS One. 2014;9:e104599. 10.1371/journal.pone.0104599 25170955 PMC4149347

[9] Rabinovich JE, Gürtler RE, Leal JA, Feliciangeli D. Density estimates of the domestic vector of Chagas disease, *Rhodnius prolixus* Stål (Hemiptera: Reduviidae), in rural houses in Venezuela. Bull World Health Organ. 1995;73:347–57. 7614667 PMC2486661

[10] Benatti RD, Oliveira GH, Bacal F. Heart transplantation for Chagas cardiomyopathy. J Heart Lung Transplant. 2017;36:597–603. 10.1016/j.healun.2017.02.006 28284779

[11] Pavão MLRC, Arfelli E, Scorzoni-Filho A, Rassi A Jr, Pazin-Filho A, Pavão RB, et al. Long-term follow-up of Chagas heart disease patients receiving an implantable cardioverter-defibrillator for secondary prevention. Pacing Clin Electrophysiol. 2018;41:583–8. 10.1111/pace.13333 29578582

[12] Stein C, Migliavaca CB, Colpani V, da Rosa PR, Sganzerla D, Giordani NE, et al. Amiodarone for arrhythmia in patients with Chagas disease: A systematic review and individual patient data meta-analysis. PLoS Negl Trop Dis. 2018;12:e0006742. 10.1371/journal.pntd.0006742 30125291 PMC6130878

